# [Corrigendum] Dihydroartemisinin attenuates osteoclast formation and bone resorption via inhibiting the NF-κB, MAPK and NFATc1 signaling pathways and alleviates osteoarthritis 

**DOI:** 10.3892/ijmm.2026.6018

**Published:** 2026-10-19

**Authors:** Dong Ding, Jiangbo Yan, Gangning Feng, Yong Zhou, Long Ma, Qunhua Jin

Int J Mol Med 49: 4, 2022; DOI: 10.3892/ijmm.2021.5059

Following the publication of the above article, a concerned reader drew to the authors' attention that one of the antibodies the authors had used in their study was apparently selected inappropriately: the Abcam antibody the authors claimed to have used to probe for phosphorylated (p)-ERK in the *Materials and methods* section (cat. no. ab229912) targets the protein kinase RNA-like ER kinase (PERK; also known as EIF2AK3), rather than p-ERK. In addition, overlapping sections of data shown in different data panels in Fig. 8A and Fig. 9B on p. 13 and p. 14 respectively, both of which showed the results of various histochemical experiments, suggested that the data in these figures had been assembled incorrectly. Specifically, the 'TRAP/Sham-operated' data panel for 4 weeks in Fig. 8A contained an overlapping section with the 'TRAP/Sham-operated' data panel for 8 weeks, and in Fig. 9B, the 'CXCL12/DMM+vehicle' data panel contained an overlapping section with the 'CXCL12/DMM+DHA' data panel. Finally, upon performing an independent analysis of the data in this paper in the Editorial Office, it came to light that the 'DMM+vehicle' data panel for 8 weeks in Fig. 8A subsequently reappeared in another article written by the same research group in the journal *PLoS One*.

Concerning the potential selection of the incorrect antibody to target p-ERK, the authors investigated their records, and realized that an error had been made in terms of writing the catalogue number for the antibody in the Materials and methods section. They provided the Editorial Office with evidence of the proof of purchase for this antibody, which in fact had the catalogue number 28733-1-AP-50 (for Phospho-ERK1/2 polyclonal antibody). Moreover, after re-examining their original data, the authors have realized that the data in Figs. 8A and 9B were assembled incorrectly. Regarding Fig. 9B, the data panel shown for the 'CXCL12/DMM+vehicle' experiment should have been included in this figure to represent the 'CXCL12/DMM+DHA' experiment, whereas the image for the 'CXCL12/DMM+DHA' experiment should have been selected from an entirely separate group of images.

Revised versions of Fig. 8 and 9, now showing the correct data for the 'TRAP/Sham-operated' data panel for 4 weeks and the 'DMM+vehicle' data panel for 8 weeks in Fig. 8A, and the correct images for the 'CXCL12/DMM+vehicle' and the 'CXCL12/DMM+DHA' data panels in Fig. 9B, are shown on the next two pages. Note that the revisions made to these figures do not affect the overall conclusions reported in the paper. All the authors agree with the publication of this corrigendum. The authors express their gratitude to the Editor of *International Journal of Molecular Medicine* for allowing them the opportunity to publish this corrigendum, and apologize to the readership for any inconvenience caused.

## Figures and Tables

**Figure 8.**
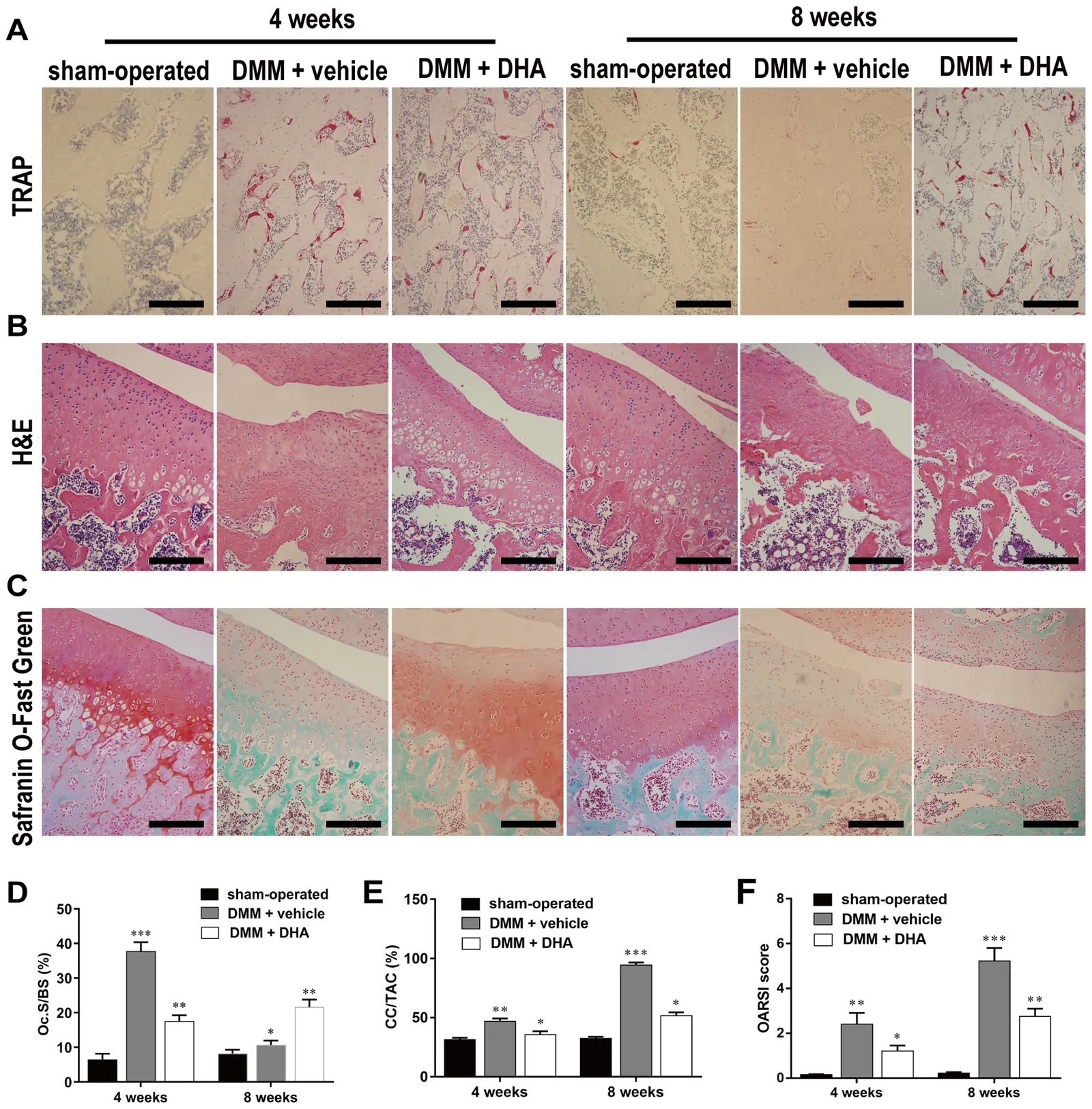
DHA suppresses cartilage degeneration by inhibiting osteoclastogenesis in the early stage of DMM-induced OA rats. The DMM-induced OA rats were treated with or without DHA for 4 and 8 weeks, and histological analysis of osteoclasts in the subchondral bone and cartilage was performed with (A) TRAP, (B) H&E and (C) Safranin O-Fast Green staining. Scale bar, 200 *μ*m. Quantitative analysis of (D) Oc.S/BS, (E) CC/TAC and (F) OARSI scores (n=6 per group/time point). ^*^P<0.05, ^**^P<0.01 and ^***^P<0.001 vs. sham-operated group. DHA, dihydroartemisinin; DMM, destabilization of the medial meniscus; OA, osteoarthritis; TRAP, tartrate resistant acid phosphatase; Oc.S, osteoclast surface; BS, bone surface; CC, calcified cartilage; TAC, total articular cartilage; OARSI, Osteoarthritis Research Society International.

**Figure 9.**
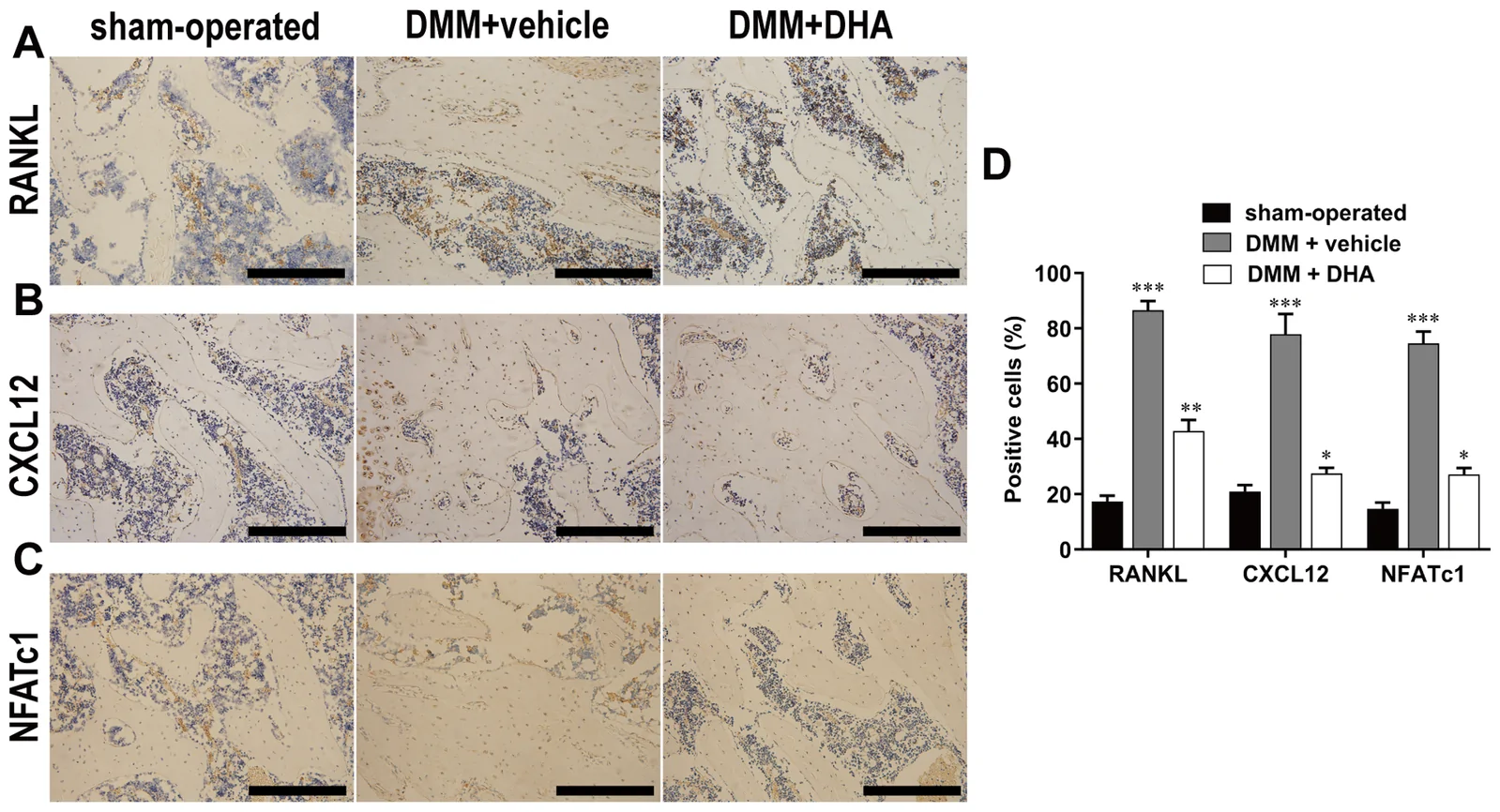
DHA attenuates the expression levels of RANKL, CXCL12 and NFATc1 during the early stage of DMM-induced OA in a rat model. The DMM-induced OA rats were treated with or without DHA for 4 weeks, and the expression levels of (A) RANKL, (B) CXCL12 and (C) NFATc1 were revealed by immunohistochemistry staining. Scale bar, 200 *μ*m. (D) Quantitative analysis of RANKL, CXCL12 and NFATc1 (n=6 per group/time point). ^*^P<0.05, ^**^P<0.01 and ^***^P<0.001 vs. sham-operated group. DHA, dihydroartemisinin; RANKL, receptor activator of nuclear factor κB ligand; CXCL12, C-X-C motif chemokine ligand 12; NFATc1, nuclear factor of activated T cell cytoplasmic 1; DMM, destabilization of the medial meniscus; OA, osteoarthritis.

